# The effect of plasticizers on rheological, physical and mechanical properties of low cement high alumina gunning refractories

**DOI:** 10.1038/s41598-025-86925-9

**Published:** 2025-01-22

**Authors:** Mohammad Mobasheri, Ali Mohammad Hadian, Yasaman Mohammadi, Behzad Azimi, Mehrnoosh Kamali

**Affiliations:** 1 School of Metallurgy and Materials Engineering, College of Engineering, University of Tehran, P.O. Box 11155-4563, Tehran, Iran; 2 The Laboratory of Research and Development, ZICO Refractories Co, Tehran, Iran

**Keywords:** Refractory, Gunning, Alumina, Plasticizers, Physical properties, Mechanical properties, Engineering, Materials science

## Abstract

In this research, the effect of different plasticizers with different amounts on the properties of monolithic alumina-based refractories has been investigated. All samples were fired at 1100 °C and 1550 °C. In order to evaluate the desired properties, first the rheological properties of the samples were examined, and then for further investigations, loss on ignition (LOI), percentage of permanent linear changes (PLC), apparent porosity (AP), bulk density (BD) and cold crushing strength (CCS) tests were used. In addition, scanning electron microscopy (SEM), energy dispersive X-ray spectroscopy (EDX) and X-ray diffractometry (XRD) were used to characterize the samples. 1 and 2 wt% of CMC, H19, bentonite and ballclay were added to the mixtures as plasticizers. The results of this research showed that the sample containing 1 wt% of ball clay can be the most appropriate one due to its highest strength, highest density and lowest apparent porosity. Moreover, the sample containing 2 wt% of H19 (a commercially available binder) has the optimum properties because of its highest strength for the samples fired at 1100 °C. For the mixtures fired at 1550 °C, the more amount of silica has caused higher cold crushing strength due to the presence of the low melting point phases which are not desired and therefore, the mixtures with less silica can be used.

## Introduction

Ceramics is a modern and diversified area which has have roots in ancient technology beginning from more than 24,000 years ago^1^. Among different categories of ceramic materials, refractory materials are defined as inert inorganic solid materials which their chemical and physical properties make them useful for structures or system components exposed to environments above 538 °C^2,3^. Refractories act as the backbone of industry since metal and steel industries are the world largest consumers of refractories^4,5^. The quality of the refractories must be improved by the refractory industries to meet the demands of other industries such as aluminum, copper, glass and cement^6^.

Refractories can be classified based on different criteria, one of which is their shape; based on this, they are divided into two categories: shaped and unshaped (monolithic) refractories^7,8^. Regarding this, unshaped refractories have received much attention in recent years^9^. The advantages of unshaped refractories include shorter production process, ease of production, increased efficiency and easier and cheaper installation^9–11^.

Among the unshaped refractories, gunning mixtures are refractories that the refractory layer must be sprayed on the surface; therefore, the paste must have certain rheological properties to provide the necessary adhesion^12^. These masses are applied under high speed and pressure in order to create compact, homogeneous and crack-free linings^13^. Gunning compounds are also very important due to their quick installation, hot gunning repair in the steel manufacturing industries, economic advantage and reducing the downtime^14–20^. These materials are widely used in metallurgy industries for relining or repairing of ladles, melting furnaces such as blast furnace, BOF furnace, electric arc furnace, tundish, and etc^21,22^. They are also used in cement industry for repairing of non-accessible sections of cyclones. This technique increases the working life of a furnace considerably and the use of appropriate gunning masses causes achieving greater hot repairing advantages^23–25^. Among gunning refractories, high alumina gunning mixtures are of high purity, working temperatures up to 1650 °C and offering dimensional stability^26^; therefore, raw materials based on tubular alumina have been used in this study.

Various binding systems can be used in refractories such as colloidal silica and resin bonding systems^13,17,27^. However, calcium aluminate cement (CAC) is widely used as a binder due to its handling complexity and also providing green strength by forming calcium aluminate hydrates in the early stages of hardening^28–30^. In this case, the reduction of CAC can be beneficial since reducing the amount of cement leads to a significant increase in the lifetime of alumina-based castables, because the lower amount of cement prevents the formation of liquid phases at high temperatures. The reason for this is related to the effect of the presence of lime in refractory materials which causes the formation of low melting temperature compounds and these compounds could deteriorate the high-temperature properties^28,31^; as a result, the amount of cement content in this study is low and it is categorized as low cement refractories.

Plasticizers are a part of gunning mixtures that are added to improve their adhesion and reduce their rebound when they are applied to a surface by spraying^32^. Plasticizers act to form a plastic mass on the gunned surface that captures the refractory particles and holds them in place until the chemical bond reacts^33^. Schmidtmeier et al.^32^ investigated the effect of GT10SG, a type of calcined alumina, and concluded that this material resulted in good rheological behavior, improved mechanical and corrosion properties, low shrinkage and high slag resistance.

Yan et al.^34^ evaluated the effect of fused spinel and fused magnesia on the properties of gunning mixtures and finally introduced the corundum-spinel sample with 6% cement and 4% magnesia as a sample with appropriate workability. Colloidal silica instead of calcium aluminate cement in spray masses was the focus of another research by Chia-Hong Chen^35^ and the results showed that spray masses with colloidal silica had desirable properties.

These studies reveal that refractories especially the gunning ones are of significant importance. However, in spite of the importance of plasticizers in gunning refractories, there is no reported work on the investigating different amounts of available and commercial plasticizers. Therefore, in the present work, the effect of different amounts of plasticizers on the rheological, physical and mechanical properties of low cement high alumina gunning refractories has been studied.

## Experimental

### Materials and preparation of samples

In this study, tabular alumina (Zhejiang Zili Advanced Materials Co. Ltd., China), Cement 70 (70 N, Union), reactive alumina (CTC20, Almatis), silica fume (Ferosilis Gharb Pars Co.), CMC (Carboxymethyl cellulose, Kimia Tehran Acid Co.), H19 (a commercial chemical binder from Zschimmer & Schwarz Co.), bentonite (Jahan Powder Co.) and ball clay (Suravajin Aghigh Co.) were used as raw materials to prepare the samples. Four types of superplasticizers were chosen in this study bases on their use in the industry, their potential to act as superplasticizer in gunning refractories and their availability. The chemical composition of the raw materials is given in Table 1 using X-ray Fluorescence (XRF). As it is seen, the plasticizers are H19 (D_50_ = 15 μm), bentonite (D_50_ = 45 μm) and ball clay (D_50_ = 105 μm). The composition of different gunning mixtures is shown in Table 2.

**Table 1:** Chemical composition of the raw materials.

Composition (wt%) Raw material	Al_2_O_3_	SiO_2_	CaO	MgO	Fe_2_O_3_	TiO_2_	Alkalies	LOI (loss on ignition)
Tabular alumina	99.20	0.20	-	-	0.10	-	0.40	0.10
Cement	69.00	0.22	28.90	0.25	0.10	0.40	0.50	0.63
Reactive alumina	99.50	0.10	-	-	0.02	0.05	-	0.33
Silica fume	1.80	81.20	0.80	0.70	6.56	-	1.50	7.44
H19	11.00	75.00	-	-	-	-	6.00	8.00
Bentonite	16.65	64.31	2.12	3.69	1.76	-	3.68	7.79
Ball clay	32.00	47.00	0.20	-	1.80	1.50	1.00	16.50

The particle size distribution of the samples was determined using Andreasen model. The Cumulative Percent Finer Than (CPFT) reads as follows:

1 $$\:CPFT={\left(\frac{D}{{D}_{L}}\right)}^{q}\times\:100$$

Where D is the particle size (in diameter), D_L_ is the largest particle size and q is the distribution coefficient^36^. For the present work, q was chosen to be 0.26 to reach the high performance behavior which means that low water consumption, suitable pumping and high fluidity will be achieved^37^.

**Table 2:** Composition of the gunning mixtures.

Sample Raw material (wt%)	Mix 1	Mix 2	Mix 3	Mix 4	Mix 5	Mix 6	Mix 7	Mix 8
Tabular alumina	78	77	78	77	78	77	78	77
Cement	8	8	8	8	8	8	8	8
Reactive alumina	12	12	12	12	12	12	12	12
Silica fume	1	1	1	1	1	1	1	1
CMC	1	2	–	–	–	-	–	–
H19	–	–	1	2	–	-	–	–
Bentonite	–	–	–	–	1	2	–	–
Ball clay	–	–	–	–	–	–	1	2

After preparing the mixtures according to Table 2, they were dry mixed at room temperature (24 °C and 35% humidity) for two minutes in a Hobart mixer and then wet mixed for three minutes by adding the appropriate percentage of water to reach the desired rheology properties (ASTM C860). Then the sample was prepared in cubic moulds with side lengths of 5 cm (ASTM C973). In order to eliminate the bubbles inside the sample, the moulds were filled layer by layer with the subsequent vibration using a vibrator table for 20 s. The samples were then placed in a humidity chamber with a relative humidity of 75% for 24 h, then they were dried at 110 °C in the oven for 24 h. After that, dried samples fired at 1100 °C and 1550 °C for 3 h.

### Physical and mechanical tests

Cold Crushing Strength (CCS) of the samples was determined using ASTM C133 standard. The following equation was used to determine the CCS of the samples:

2 $$\:{\sigma\:}_{CCS}=\frac{{F}_{max}}{{A}_{0}}$$

Where $\:{\sigma\:}_{CCS}$ is CCS (N/mm^2^), F_max_ is force at the fracture (N) and A_0_ is surface area (mm^2^).

Apparent Porosity (AP) and Bulk Density (BD) were measured according to ASTM C20 standard using formulas:

3 $$\:AP\:\left(\%\right)=\frac{W-D}{W-S}\times\:100$$

4 $$\:BD\:\left(\frac{g}{{cm}^{3}}\right)=\frac{D}{W-S}$$

Where D is dry weight, S is suspended weight and W is saturated weight of the test piece.

The percentage of permanent linear change (PLC) was calculated according to the ASTM C179 standard that the dimensions of the samples were measured before and after firing, and then the percentage of PLC was calculated. Loss on ignition (LOI) of the samples was measured using the following equation:

5 $$\:LOI\left(\%\right)=\frac{{M}_{f}-{M}_{0}}{{M}_{0}}\times\:100$$

Where M_f_ is the final mass of the sample after heating and M_0_ is the initial mass of the sample before heating.

The absolute uncertainties for CCS, AP, BD, PLC and LOI are 5 MPa, 1%, 0.05 g/cm^3^, 0.1% and 1%, respectively.

### Characterization

Chemical analysis of the raw materials and mixtures was investigated by X-ray fluorescence (ICP-OES agilent735). A PHILIPS X-ray diffractometer with Copper K-α radiation with a voltage of 50 kV was used for phase analysis. A TESCAN scanning electron microscopy (SEM) equipped with RONTEC energy dispersive spectroscopy (EDS) detector were used for revealing the microstructures and chemical analysis.

### Rheological test

To evaluate adhesion at the laboratory level, the same method used by Peng et al.^38^ is performed. To summarize, after the sample was taken out from the Hobart mixer, a ball in hand was made and thrown towards a metal plate with some anchors or towards a glass plate. If it sticks, it passes the test; if it slips, it fails. In this study, this method was modified using the concept introduced by Gasillon et al.^39^. In other words, instead of spraying the sample on just one plate, 10 samples were thrown on 10 individual square plates having the length of 30 cm. After this, two important parameters were measured naming rebound rate (R) and spreading behavior (S). To determine the rebound rate, the average rebound rate of all samples were calculated using the following formula:

6 $$\:R\left(\%\right)=\frac{{M}_{f}}{{M}_{0}}\times\:100$$

Where M_0_ is the initial mass of the sample and M_f_ is the mass of the rebounded sample after hitting the plate.

To study spreading behavior of the samples, the covered areas of the squares were measured as surface fractions meaning that for each sample, the number of fully completed, half and three quarters covered squares were determined.

It should be mentioned that for each set of experiment three samples were prepared and examined.

## Results and discussion

### Rheological properties

To study rheological properties, several important parameters should be investigated. All the samples had an appropriate level of adhesion since all the added plasticizers are well-known commercial adhesives and can provide some level of adhesion. In this case, the focus of this research is on investigating rebound rate (R) and spreading behavior (S). The samples with the lower amount of R and more complete covered surfaces, are considered as the most appropriate ones.

Table 3 shows rebound rate (R) and spreading behavior (S) of the mixtures. As can be seen, although Mix 1 and Mix 2 has appropriate amounts of rebound, their spreading behavior is not suitable since most of these mixtures could not covered the whole surface. On the other hand, Mix 3 is an appropriate mixture regarding its spreading behavior, but it has a relatively high amount of rebound (21%). Other mixtures, from Mix 4 to Mix 8, are suitable in terms of both the rebound rate and spreading behavior. Therefore, the first three mixtures failed in terms of rheological properties. However, the others passed and they will be investigated more to choose the optimum mixtures among them. Table 3. Rebound rate (R) and spreading behavior (S) of the mixtures.Table 3. Rebound rate (R) and spreading behavior (S) of the mixtures.

**Table 3:** Chemical composition of the raw materials.

Mixtures		Mix 1	Mix 2	Mix 3	Mix 4	Mix 5	Mix 6	Mix 7	Mix 8
R (%)		16	17	21	15.5	16	16.5	17	17.5
S (%)	1/2 area	40	50	30	10	10	20	10	20
	3/4 area	40	40	10	10	20	10	30	20
	Complete area	20	10	60	80	70	70	60	60

**Fig. 1:**
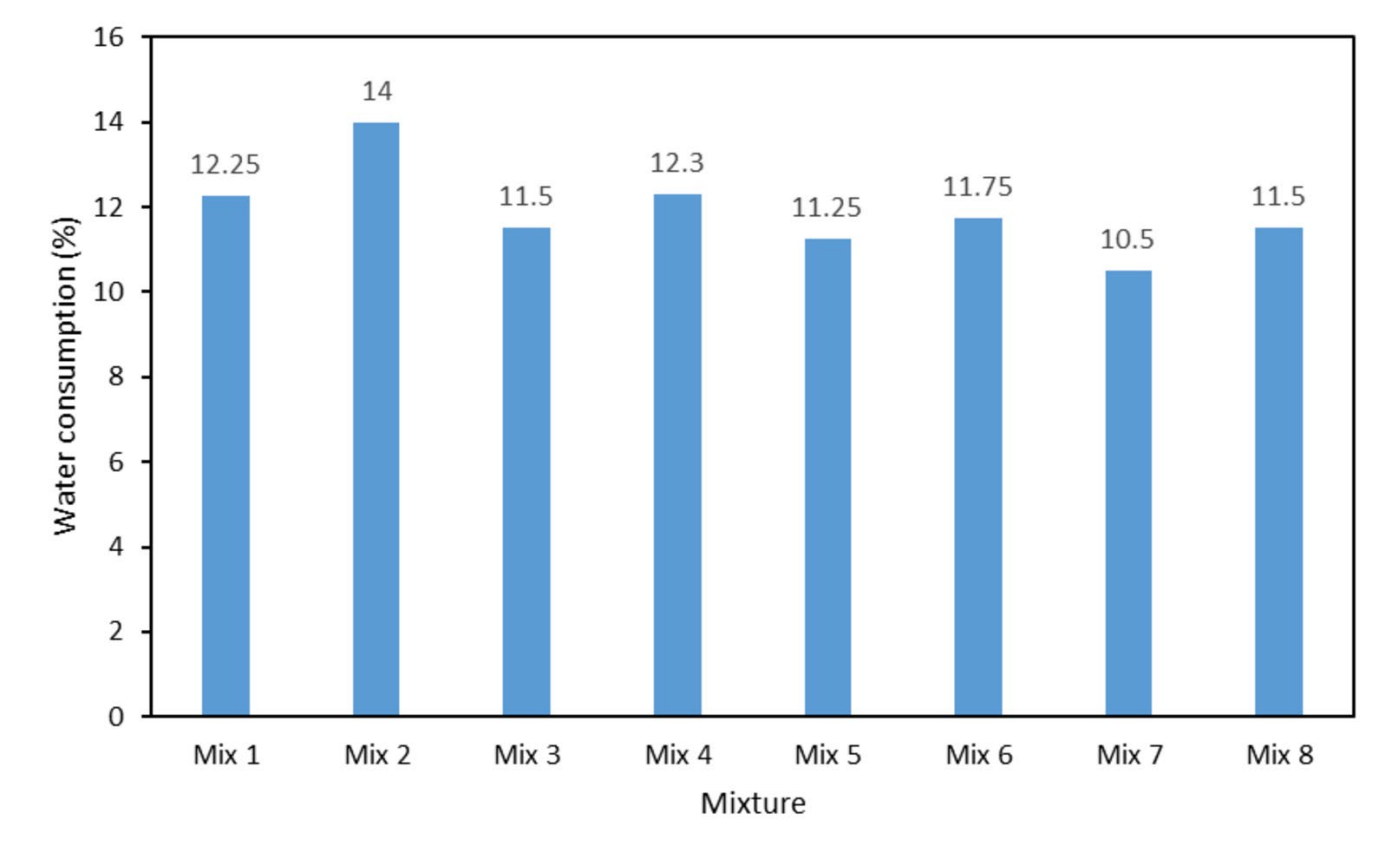
Water consumption of different mixtures.

Figure 1 shows the amount of water consumed for preparation of different mixtures. The % of water consumption is the amount of water added to the sample to reach the desired rheological properties. As can be seen, Mix 4 has the highest amount of water consumption (among the chosen samples), followed by the composition with 2% bentonite (Mix 6) with 11.75% water consumption. After that, the samples with 2% of ball clay (Mix 8) and one% of bentonite (Mix 5) has 11.5 and 11.25% of water consumption, respectively. Finally, Mix 7 has the lowest amount of water consumption.

The reason for the difference in the amount of water consumed can be related to the particle size distribution of the mixtures particularly that of the plasticizer. Therefore, H19 (D_50_ = 15 μm) with the smallest particle size, consumed the maximum amount of water and ball clay (D_50_ = 105 μm) with the largest particle size, consumed the minimum amount of water. On the other hand, the amount of water consumption is also related to the nature of the plasticizers. This means that the amount of water consumed in samples containing bentonite is higher due to the three-layered nature of this plasticizers; whereas, ball clay has a two-layered nature and requires less water compare to bentonite. The effect of the amount of water consumption on properties will be discussed later.

### Chemical analysis of the mixtures

Table 4 represents the chemical composition of the mixtures. H19, bentonite and ball clay contain highest to lowest amount of silica respectively according to Table 1. Therefore, Mix 4 contains the highest and Mix 7 contains the lowest amount of silica. Furthermore, the amount of CaO in all samples is less than 2.5% and because of this, they are considered as low cement refractories. Table 4. Chemical composition of the mixtures.Table 4. Chemical composition of the mixtures.

**Table 4:** Chemical composition of the mixtures.

Composition (wt%) Sample	Al_2_O_3_	SiO_2_	CaO	MgO	Fe_2_O_3_	TiO_2_	Alkalies
Mix 4	94.08	2.50	2.32	0.03	0.15	0.03	0.49
Mix 5	94.95	1.52	2.41	0.04	0.17	0.04	0.41
Mix 6	94.06	2.05	2.50	0.05	0.19	0.04	0.43
Mix 7	95.17	1.42	2.32	0.03	0.17	0.05	0.38
Mix 8	94.50	1.84	2.33	0.03	0.19	0.07	0.39

### Characterization

**Fig. 2:**
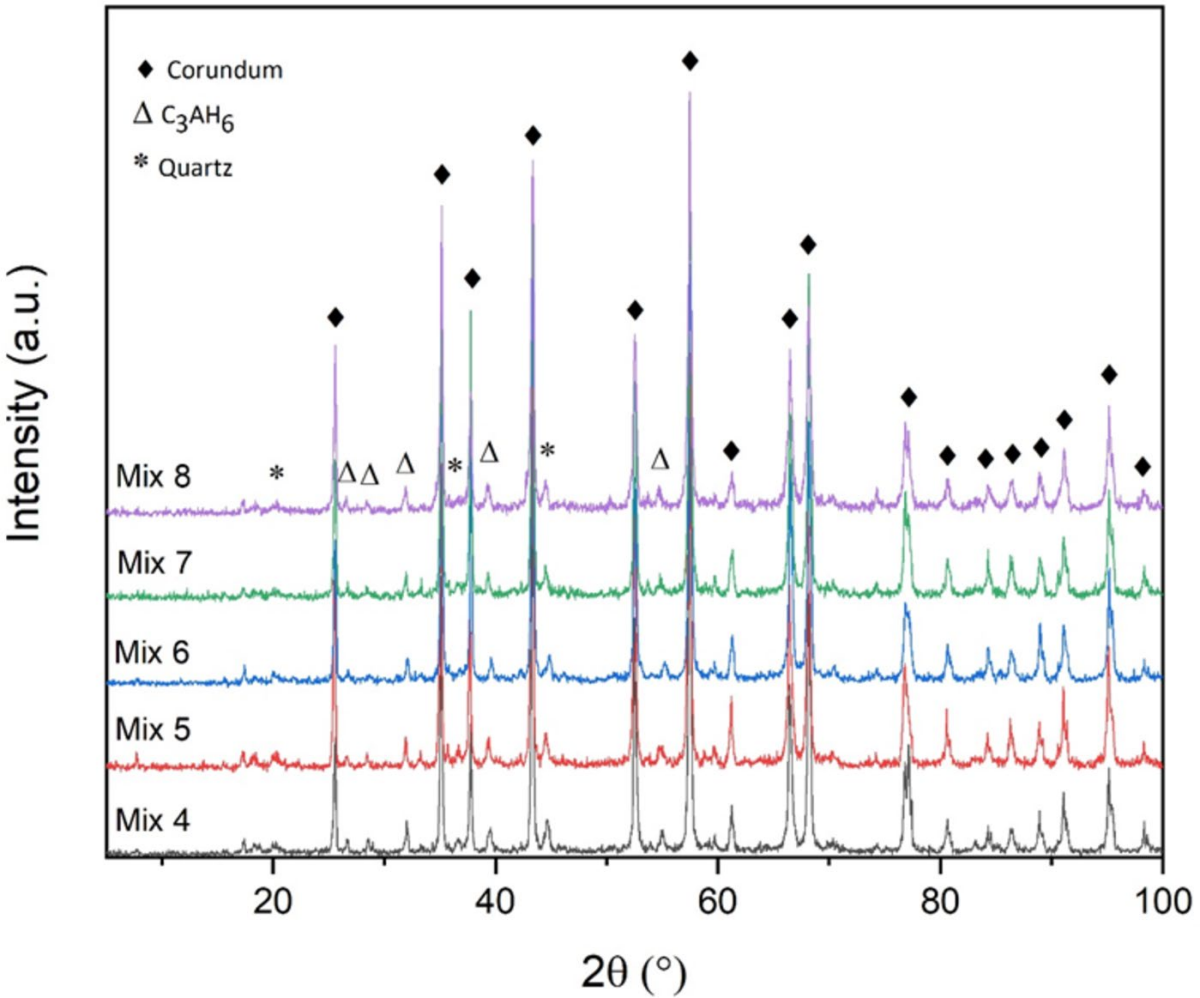
XRD patterns of the samples dried at 110 °C.

Figure 2 shows the X-ray diffraction image of the samples dried at 110 °C. As can be seen, there are corundum phase peaks in all samples, because alumina is the main component of all mixtures. As it is visible, C_3_AH_6_ and quartz phases are seen at this temperature. The C_3_AH_6_ phase is a hydrated phase that is created as a result of hydration of the cement in the samples. The presence of quartz phase in the mixtures at this temperature is also a result of the presence of SiO_2_ in the samples.

**Fig. 3:**
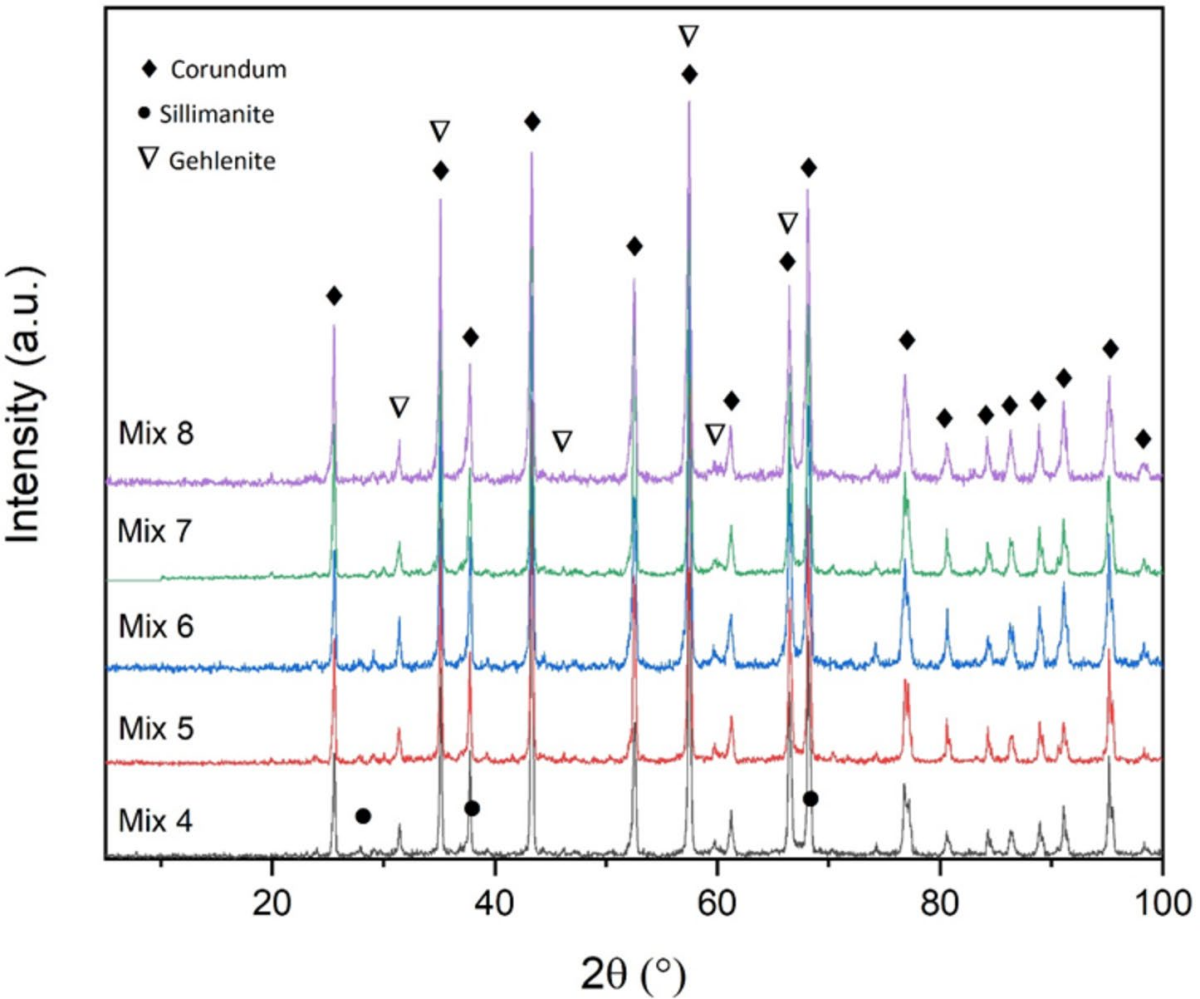
XRD patterns of the samples fired at 1100 °C.

Figure 3 shows the X-ray diffraction pattern of the samples fired at 1100 °C. At this temperature, the hydrated phase at 110 °C is dehydrated and decomposed, leading to the formation of other phases at this temperature. At 1100 °C, the gehlenite phase is formed in all samples, which is a combination of alumina, silica and calcia with chemical formula of Ca_2_Al_2_SiO_7_. sillimanite phase with chemical formula of Al_2_SiO_5_ can also be seen in Mix 4. The reason for the presence of the sillimanite phase in this sample can be related to the presence of a higher amount of silica in this sample (based on Table 4) compared to the rest of the samples, because this phase is a combination of alumina and silica. It should be mentioned that the corundum is the main phase in all samples.

**Fig. 4:**
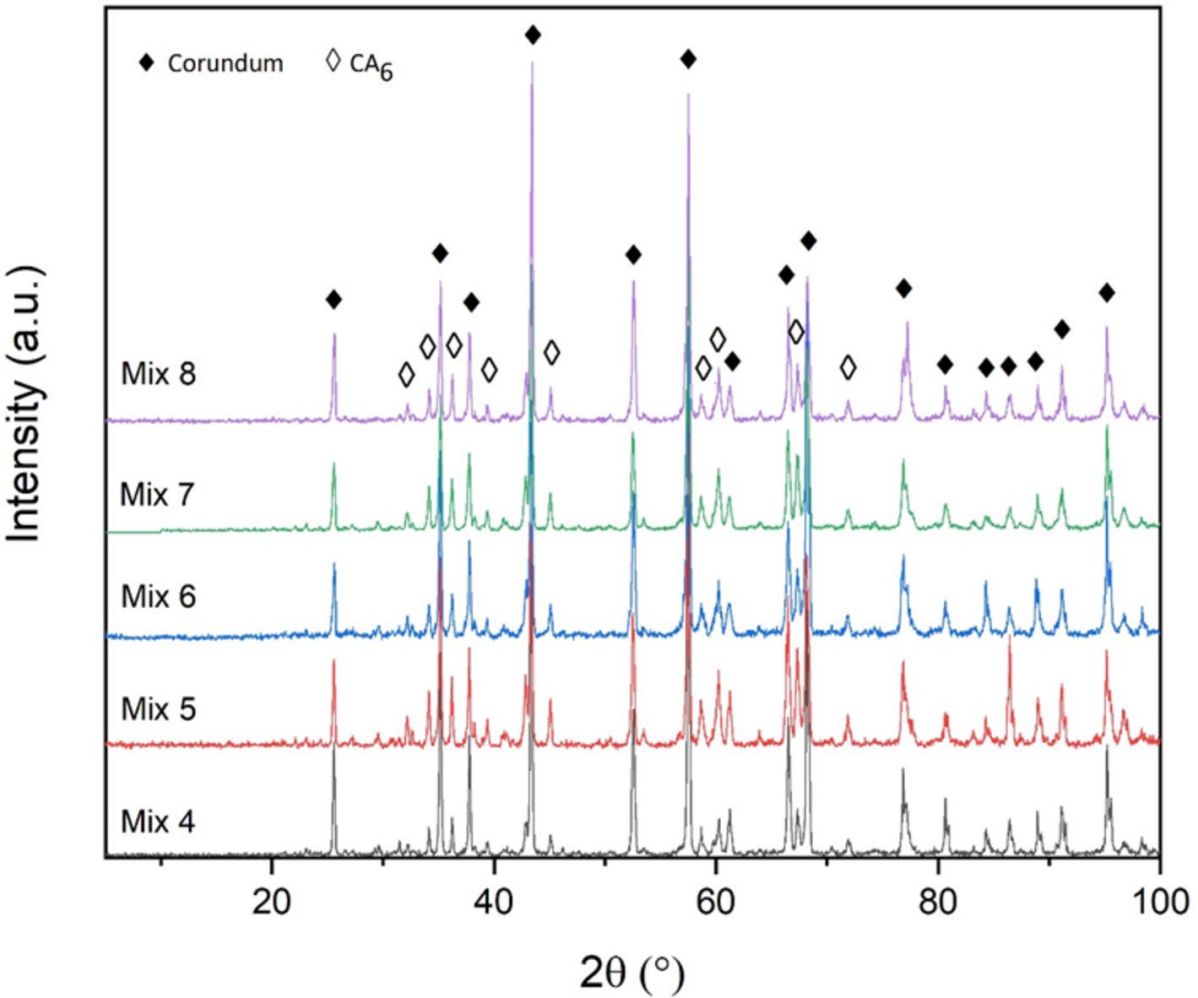
XRD patterns of the samples fired at 1550 °C.

Figure 4 shows the X-ray diffraction patterns of the samples fired at 1550 °C. As can be seen, the CA_6_ phase is the main component in all samples, along with the corundum phase.

**Fig. 5:**
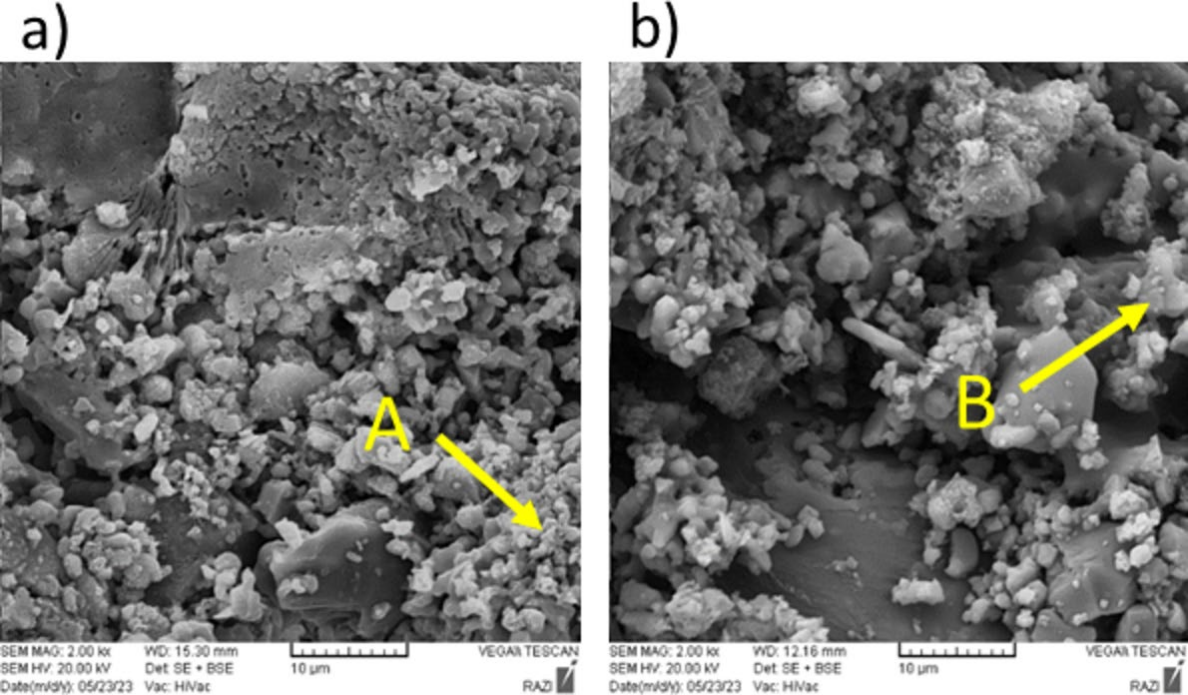
SEM images of Mix 4 fired at 1100 °C: (**a**) containing point A (gehlenite), (**b**) containing point B (sillimanite).

**Fig. 6:**
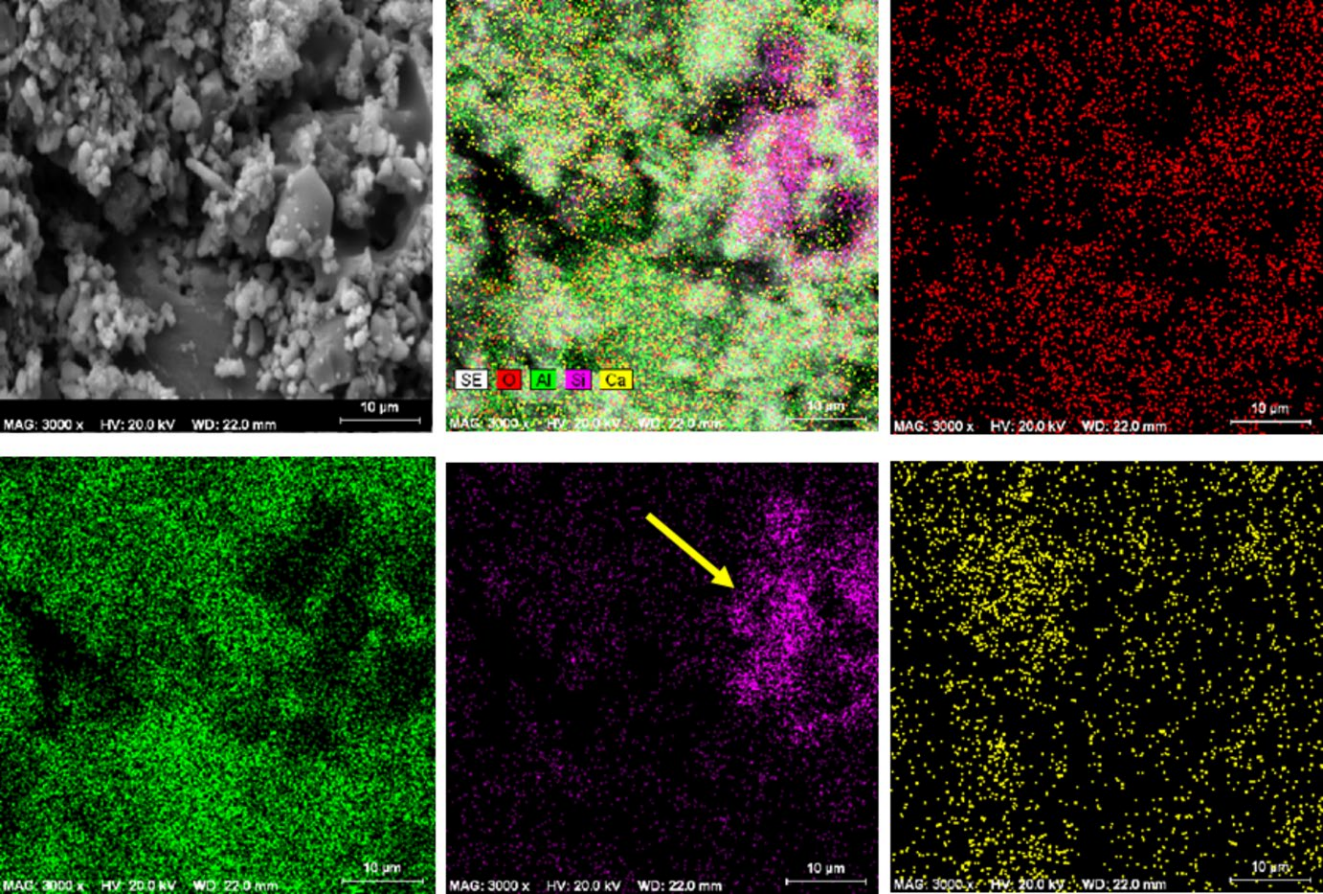
EDS map analysis of Mix 4 fired at 1100 °C.

**Fig. 7:**
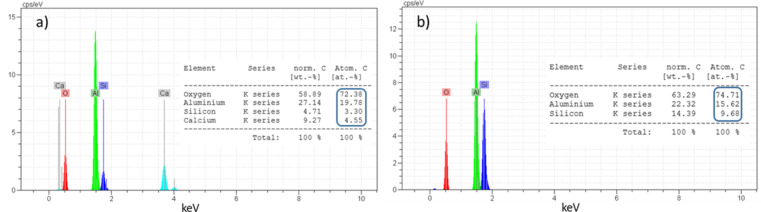
EDX spot analysis of Mix 4 fired at 1100 °C taken from: (**a**) point A and (**b**) point B.

Figure 5 shows the SEM images of Mix 4 fired at 1100 °C. EDS map analysis has been used to detect the distribution of existing elements (Fig. 6). For this purpose, EDS map analysis of Fig. 5b is shown in the Fig. 6. As can be seen, the marked part contains higher amounts of silica. Therefore, EDX point analysis has been used to quantitatively investigate these areas and identify the corresponding phases (Fig. 7). According to the Fig. 7, it can be seen that point A in the SEM image is the gehlenite phase (based on the atomic analysis). Figure 7 also confirms the presence of sillimanite phase (point B) which includes alumina and silica. It is worth mentioning that at this temperature, silica cannot melt and therefore, the available silica forms other crystalline phases, as mentioned above, by the combination with other elements.

**Fig. 8:**
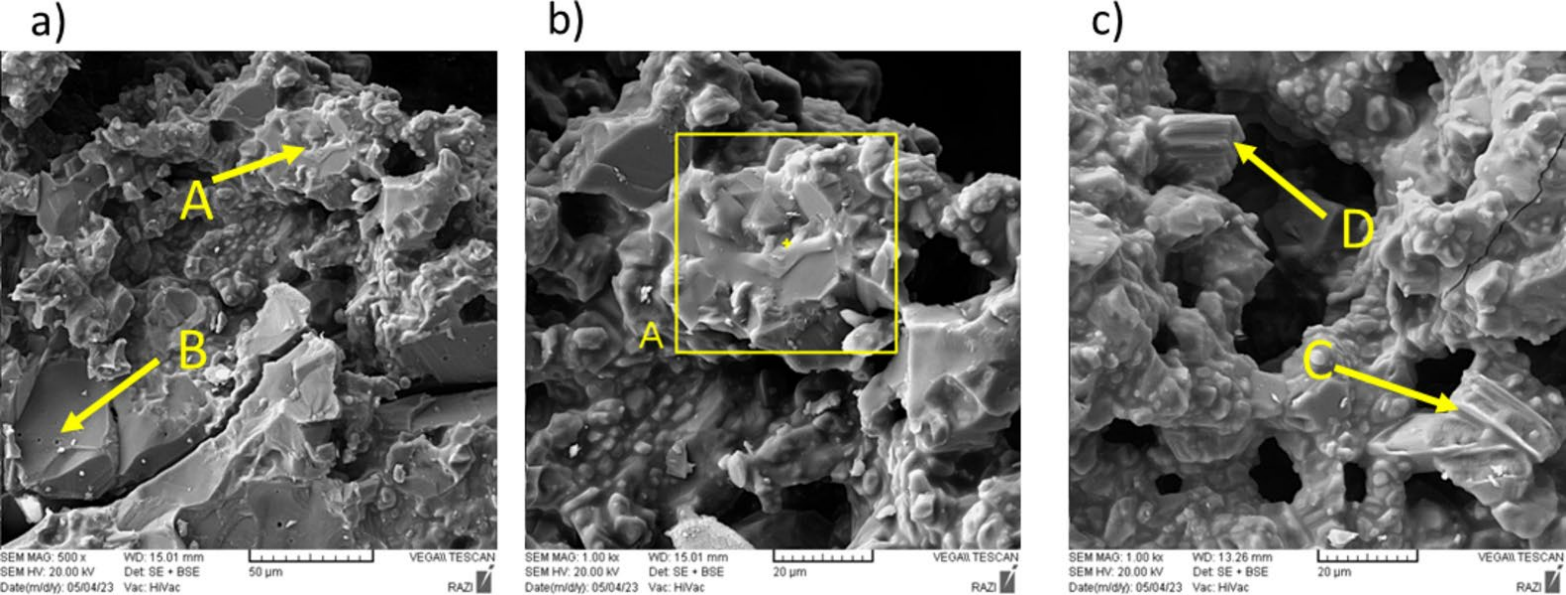
SEM images of Mix 4 fired at 1550 °C: (**a**,**b**) containing points A (amorphous phase) and B (corundum) and (**c**) containing points C (CA_6_) and D (CA_6_).

**Fig. 9:**
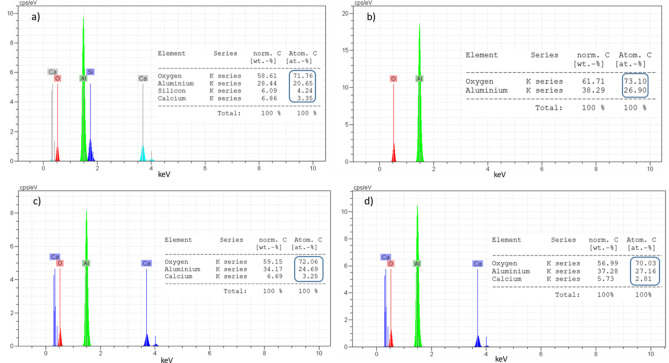
EDX spot analysis of Mix 4 fired at 1550 °C taken from: (**a**) point A, (**b**) point B, (**c**) point C and (**d**) point D.

Figure 8 shows scanning electron microscope (SEM) images of Mix 4 fired at 1550 °C. EDX spot analysis has been used to quantitatively check the composition of the points marked in the previous figure and to identify the corresponding phases. As seen in Fig. 9a, point A contains all the available elements (Al, Si, Ca, O). Considering the shape of this phase in the SEM images and also the absence of this phase in the X-ray diffraction patterns, it can be concluded that this phase is amorphous. The same incident leads to difficulty in observing the remaining phases such as the CA6 phase. The reason for not seeing the hill in the XRD patterns is also due to the low amount of this phase, which of course is spread on the surface of the particles in all the samples. This point can be understood from the EDS map images that, in addition to aluminum and oxygen, which are the main elements, silicon and calcium elements are present in a significant part of the sample, although in small amounts. In fact, the compounds containing silica available in the samples, melt at higher temperatures (1550 °C) and as a result, dissolves other phases such as CA6 in itself; as a consequence, the amorphous phase in which all the mentioned elements exist, is seen in the SEM images and is detected by the EDX point analysis.

The presence of alumina particles (corundum phase) in the sample can also be according to Fig. 9b, which can be matched with the corresponding SEM image. Also, based on Fig. 9c and d, it is possible to confirm the presence of CA6 phase in the sample, which is in good agreement with the atomic percentages related to this phase. The CA6 phase is generally in the shape of a hexagonal cylinder; a cross-section of this phase can be seen in the Fig. 8c. It should be noted that the phases seen in the SEM images and EDX point analysis taken from them are in a good agreement with the results of XRD analysis.

**Fig. 10:**
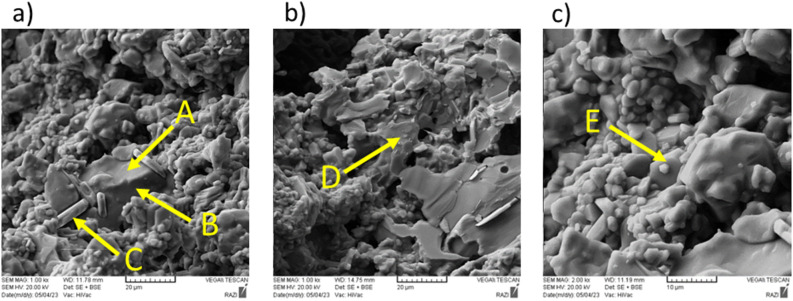
SEM images of Mix 7 fired at 1550 °C: (**a**) containing points A (corundum), B (amorphous phase) and c (CA_6_), (**b**) containing point D (amorphous phase) and (**c**) containing point E (CA_6_).

**Fig. 11:**
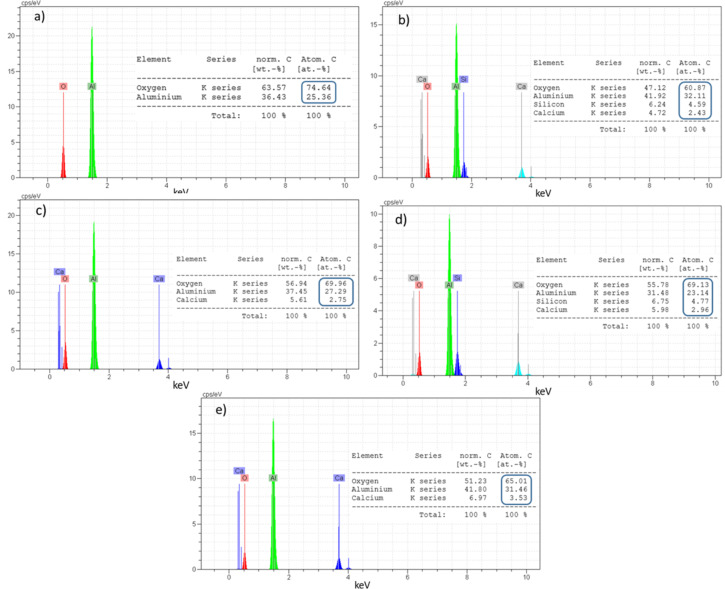
EDX spot analysis of Mix 7 fired at 1550 °C taken from: (**a**) point A, (**b**) point B, (**c**) point C, (**d**) point D and (**e**) point E.

Figure 10 shows the SEM images of Mix 7 fired at 1550 °C. The EDX analysis of the corresponding points in Fig. 10 are shown in Fig. 11. As seen in Fig. 11a, point A is an alumina particle (corundum phase). Figure 11b shows point B, which is the amorphous phase. According to the shape of this phase in the SEM images and the absence of this phase in the X-ray diffraction images, we can conclude that this phase is amorphous and forms amorphously on the surfaces of other particles such as alumina. On the other hand, in addition to the mentioned cases, another reason for the amorphous nature of this phase can be found in Fig. 10 and points A and B. In this case, at first glance, the observed particle seems to be only alumina particle, but as it is clear in EDX analysis, part of it is alumina and another part contains all elements. Therefore, it can be seen that the amorphous phase is formed on the alumina particle and has an amorphous state. Figures 10b and 11d also show another image and analysis of this amorphous phase, which is consistent with the previous statements.

Figure 11c and e respectively show the analysis of points C and E in Fig. 10. Based on the analysis of these points, it can be seen that the specified particles are the CA6 phase, which is evident according to the atomic percentages of the analysis. Also, the shape of these particles is a hexagonal cylinder, which can be seen laterally in Fig. 10c and the cross section of this phase is visible in Fig. 10a. Also, in this sample, due to the lower amount of silica (based on Table 4), CA6 particles, as seen in the SEM images, can be seen more easily than Mix 4, which had a higher amount of silica.

### Physical properties

#### Loss on ignition (LOI)

**Fig. 12:**
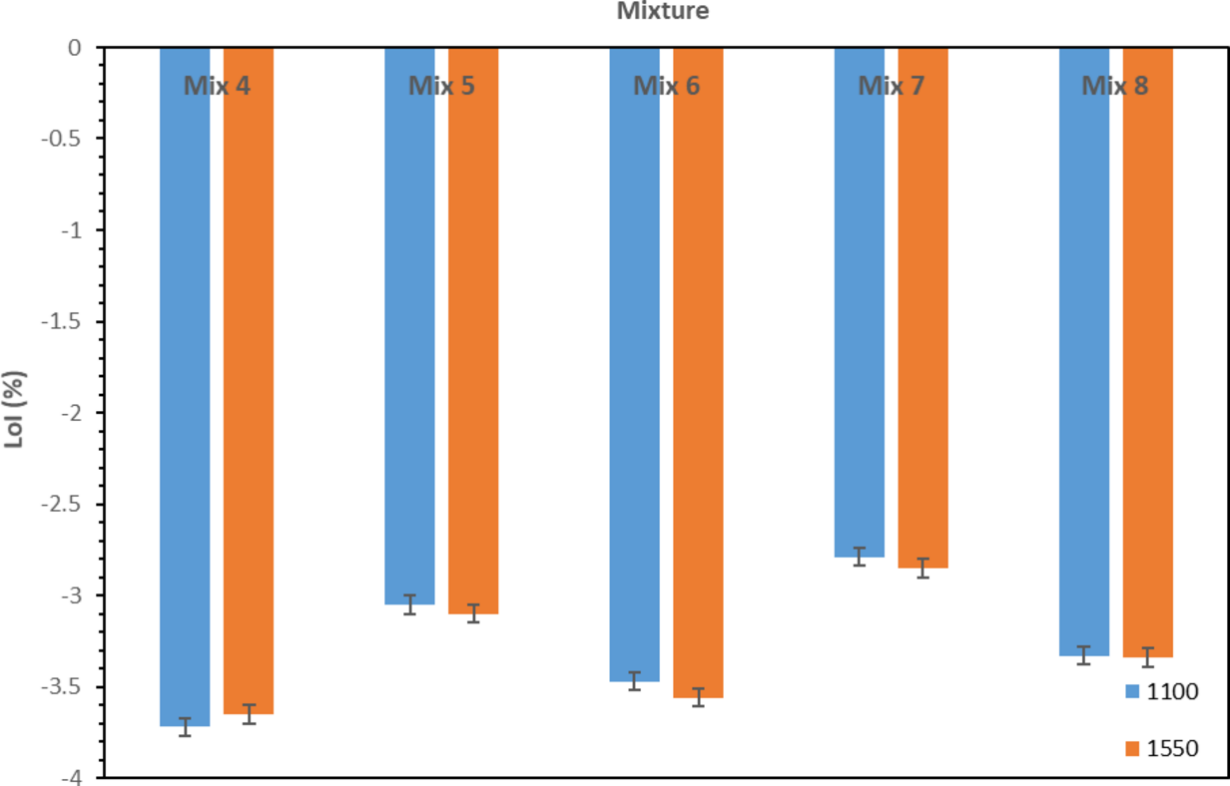
Loss on ignition for different mixtures.

Figure 12 shows the changes in the mass of the samples (i.e. loss on ignition (LOI)) with different compositions as a result of firing the samples at 1100 °C and 1550 °C. According to the figure, it can be understood that the LOI for all 5 compounds is negative, which means that due to the increase in temperature from 110 °C to 1100 °C and 1550 °C, all the samples have lost their structural water and as a result their mass decreased. The observed mass reduction is primarily attributed to the removal of water from the samples, where most volatile substances are released. Other volatile substances play a less important role as the content of water consumption is large and also controlling. It should also be mentioned that the amount of mass changes in two temperatures for each composition is almost equal because up to 1100 °C, almost all the volatile substances have been removed and above this temperature there will not be much changes in the mass of the samples.

As can be seen in the figure, the sample with a higher amount of water consumption (Fig. 1) experienced a greater percentage of mass reduction after heating from 110 °C to 1100 °C. In this way, the existing pattern is the same as the graph of the amount of water consumed, and Mix 4 has the highest percentage of mass reduction whereas Mix 7 has the lowest percentage of this amount.

#### Permanent Linear Change (PLC)

**Fig. 13:**
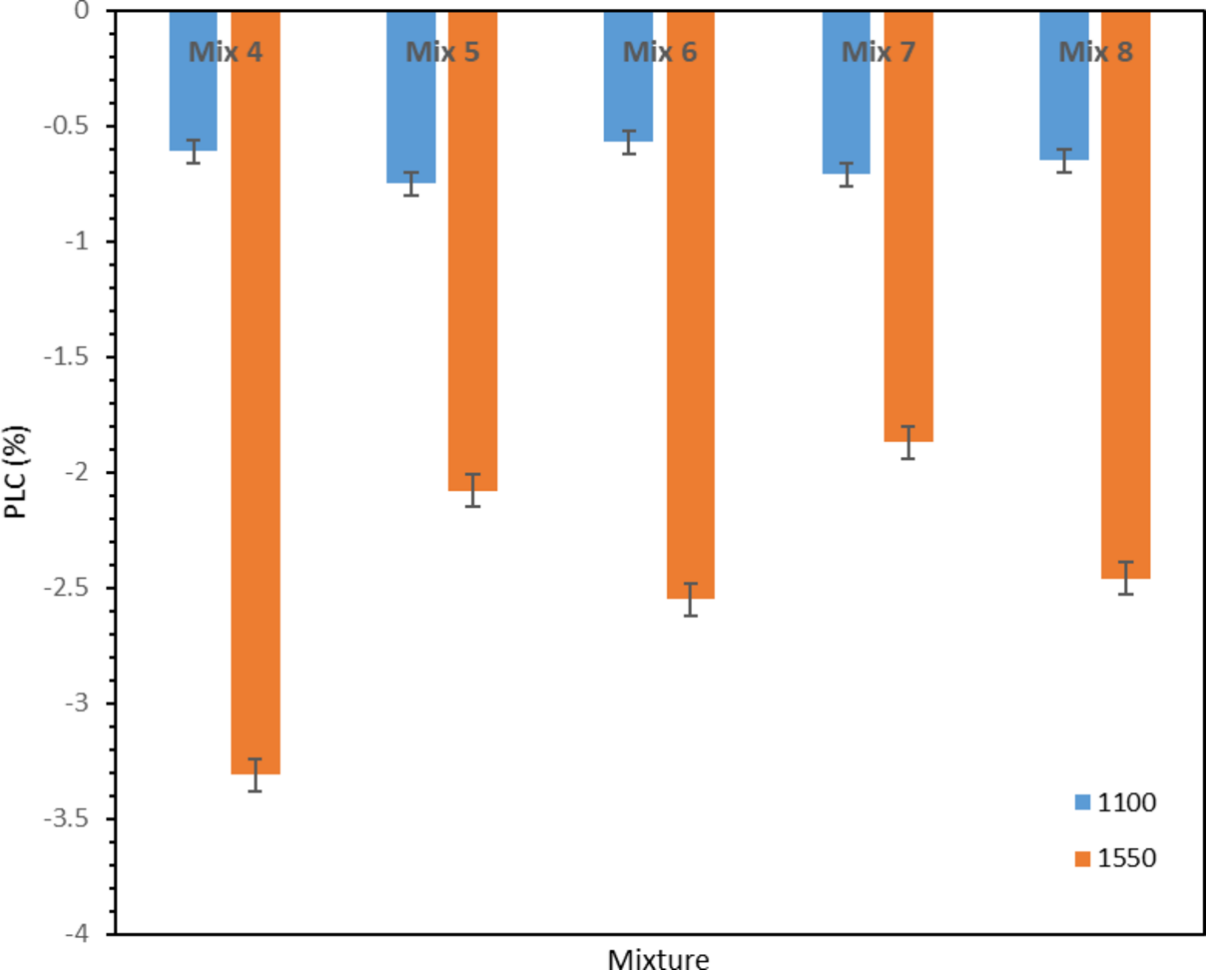
PLC (%) of different mixtures.

Figure 13 shows the amount of permanent linear changes (%) versus the chemical composition of the samples fired at 1100 °C and 1550 °C. As can be seen in this figure, all the compounds fired at 1100 °C have negative PLC, indicating occurrence of shrinkage in these samples. At this temperature, gehlenite is formed in all samples according to the XRD patterns. The formation of this phase is accompanied by expansion^40,41^ but as can be seen in the figure, all the samples have experienced contraction at 1100 °C, which indicates that the sintering process of the samples has started at this temperature. Therefore, the two factors of sintering and the formation of gehlenite phase neutralize each other as much as possible and finally, as can be seen, all the samples have almost equal and low negative dimensional changes at this temperature.

For the samples fired at 1550 °C, as can be seen in Fig. 13, all the samples have shrinkage and the PLC of the samples are negative values. At this temperature, the amount of shrinkage is higher than at 1100 °C, because at 1550 °C, the sintering process takes place, which leads to a sharp reduction in dimensions. As it is clear, by comparing the samples containing 1% of bentonite and ball clay, it is determined that Mix 5 has more shrinkage due to the higher amount of silica (based on Table 4), and its PLC is -2.08; while PLC of Mix 7 is -1.87. The effect of higher amount of silica on the increase of shrinkage at this temperature is due to the fact that by increasing the amount of silica, more amorphous phase is formed, and this phase dissolves more surrounding particles and thus leads to a further decrease in dimensions.

At 1550 °C, it is clear by comparing the Mix 4, 6 and 8 that the more amount of silica leading to more shrinkage according to the reason explained above. Thus, Mix 4, with the highest amount of silica (based on Table 4), has the highest shrinkage, followed by Mix 6 and finally Mix 8 has the lowest shrinkage.

#### Apparent porosity (AP) and bulk density (BD)

**Fig. 14:**
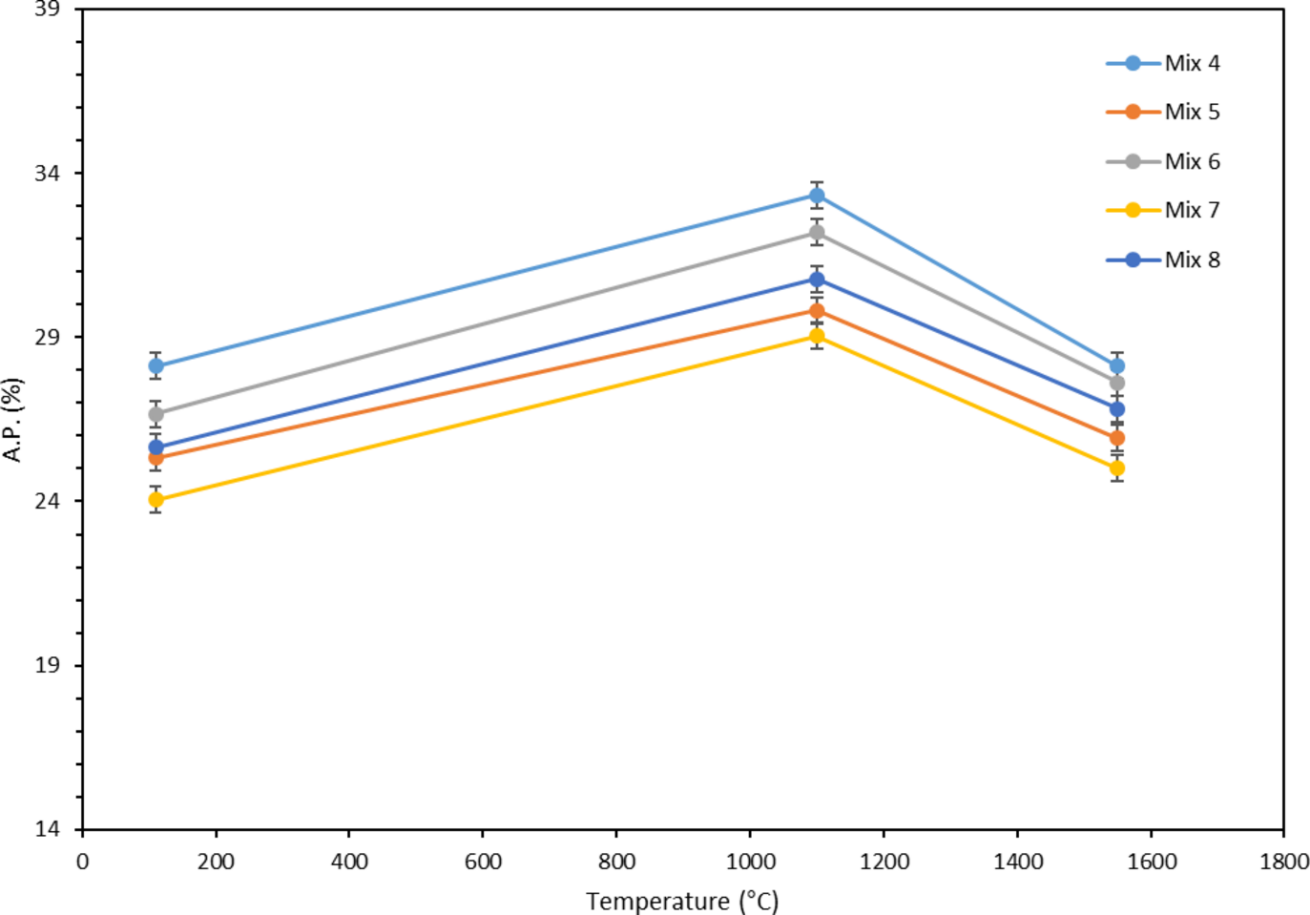
Apparent porosity versus temperature for different mixtures.

Figure 14 shows the percentage of apparent porosity in terms of temperature for different mixtures. As it is clear from the figures, in general, apparent porosity increases with increasing temperature from 110 °C to 1100 °C. The reason for this can be explained by the exit of water at 1100 °C (according to Fig. 12), which leaves porosity. It should be noted that at this temperature, according to Fig. 13, sintering of the samples is also observed, which is a reason for the reduction of apparent porosity, but the effect of the reduction in mass exceeds the reduction of the dimensions of the samples, and the overall percentage of apparent porosity at 1100 °C increases. Further increasing the temperature to 1550 °C leads to the reduction in apparent porosity. At this temperature, as explained earlier, the amount of mass reduction is almost equal to that of samples fired at 1100 °C. Therefore, with the increase in temperature from 1100 °C to 1550 °C, there is no significant decrease in the mass of the samples, but according to Fig. 13, at 1550 °C, the severe contraction of the samples takes place, which leads to the closing of the pores (due to the sintering and also formation of the amorphous phase) and, as a result, the amount of apparent porosity decreases. It is also worth mentioning that the melting of the compounds containing silica and dissolving the surrounded phases can also result in the contraction of the samples and also filling the pores.

As can be seen in Fig. 14, at 110 °C, Mix 7 has the lowest percentage of apparent porosity (24%) and Mix 4 has the highest amount (28%). Mix 5, 8 and 6 also have 25, 26 and 27% apparent porosity, respectively. As it is known, the trend observed in the percentage of apparent porosity is similar to the amount of water consumption; in this way, Mix 7 with the lowest amount of water consumption has the lowest apparent porosity and Mix 4 with the highest amount of water used has the highest amount of apparent porosity. This process is true for the rest of the mixtures in the same way. The reason for this is that the sample with more water consumption, at 110 °C, experienced more water (structural water) outflow leading to an increase in apparent porosity.

At 1100 °C, the order of percentage of apparent porosity of all samples is the same as 110 °C; this means that, for example, Mix 7 has the lowest (29%) and Mix 4 has the highest (33%) apparent porosity. At this temperature, as seen in Fig. 12, Mix 4 has the highest mass change (-3.72%) and as a result, the amount of water released is higher and its apparent porosity is also the highest. On the other hand, Mix 7 has the lowest amount of mass changes (-2.79%), its water release rate is lower, and finally its apparent porosity is the lowest among the samples. The same argument is true for the rest of the mixtures, so that Mix 5, 6, and 8, which have LOI of 3.05, -3.47, and − 3.33, respectively, their apparent porosity is equal to 30, 32, and 31%, respectively. Another important point in this regard is that all the samples have contracted at this temperature according to Fig. 13, but because the contraction of all the samples is almost equal to each other, therefore the effect of mass changes is greater and as a result, the apparent porosity of the samples depends on LOI.

Almost the same behavior is observed for the samples fired at 1550 °C in which Mix 7, 5, 8, 6 and 4 have the lowest to the highest porosity. At this temperature, according to Fig. 12, the amount of LOI for all samples is the same as at 1100 °C and follows the same trend. Therefore, the sample with a higher LOI has a higher apparent porosity percentage, which is confirmed by the apparent porosity percentages of the samples fired at 1550 °C. On the other hand, according to Fig. 13, the mixtures at this temperature have a more intense contraction than at 1100 °C, and this contraction should have an effect on the apparent porosity of the samples, but as seen in Fig. 14, the effect of mass changes is still more than dimensional changes and as a result, the samples follow the trend of mass changes. It should be noted that this severe shrinkage of the samples has led to a decrease in the range of changes in the apparent porosity of different mixtures at 1550 °C compared to 1100 °C.

**Fig. 15:**
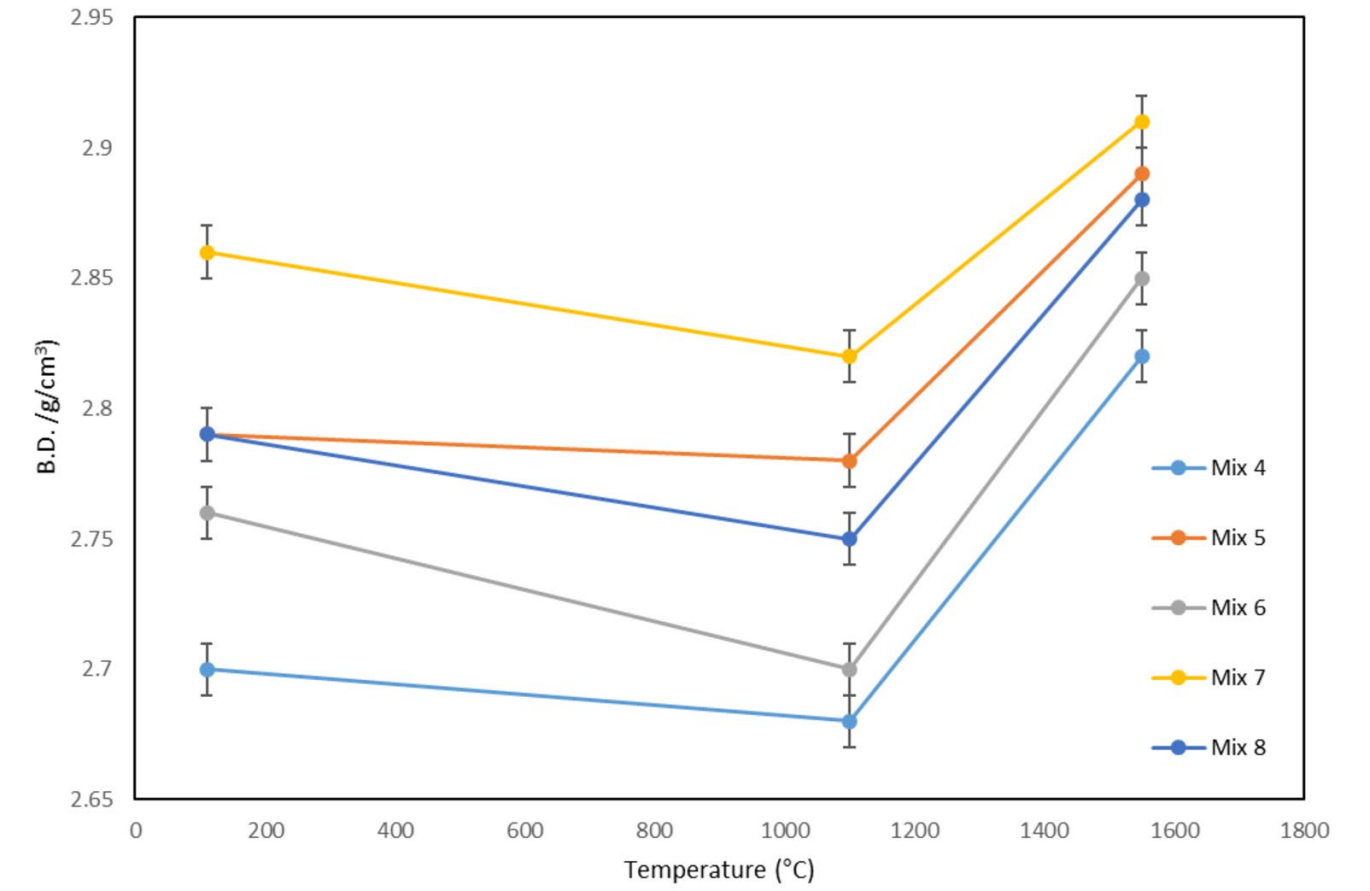
Bulk density versus temperature for different mixtures.

Figure 15 shows the density changes versus temperature for different mixtures. As it is seen, these two parameters show opposite behavior. In fact, in relation to the density of the samples at different temperatures and for different compositions, the arguments mentioned in the previous section about apparent porosity are valid and are also true in this case. To mention briefly, at 110 °C and 1100 °C, the main reason for density trend and change is related to the LOI and the exit of water which reduces the mass of the samples and the density. Furthermore, at 1550 °C, the contraction of the samples plays an important role in increasing density.

Comparing the results of this study with^32^, the bulk density results are superior. Moreover, the apparent porosity results of the current study are comparable with the mentioned research at 110 °C and 1100 °C; but, superior at 1550 °C. Therefore, the physical properties are completely accepted for this type of refractory.

### Mechanical properties

**Fig. 16:**
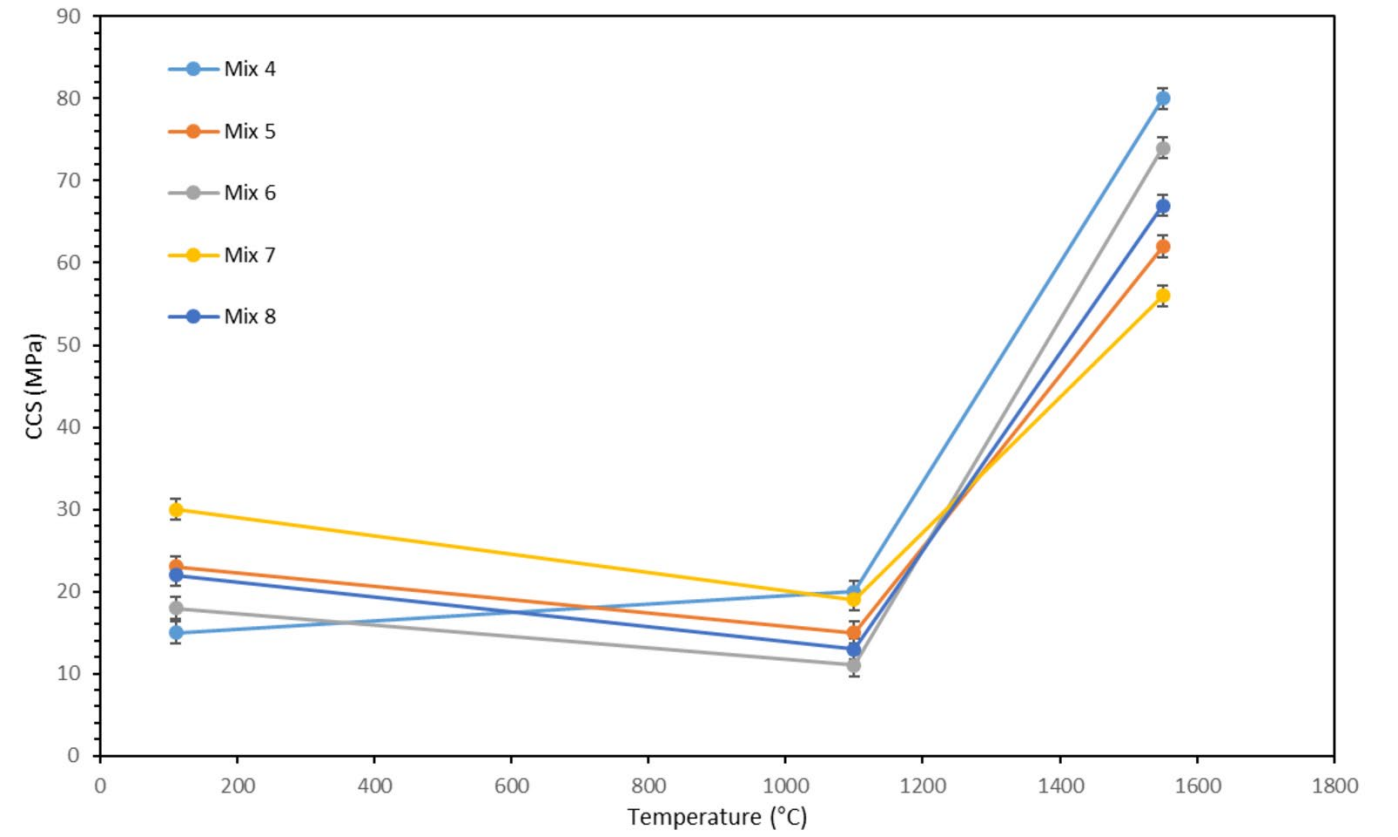
Cold crushing strength versus temperature for different mixtures.

Figure 16 shows variation of cold crushing strength versus temperature for different mixtures. As can be seen in the figure, at 110 °C, the strength of the samples is consistent with the amount of water consumed; in such a way that the composition with more water consumption has lower strength due to higher porosity. On the contrary, the composition with less water consumption has more strength. In fact, the changes in the strength of the samples are in accordance with the changes in their density. In this regard, it can be generally said that an increase in apparent porosity in a sample leads to a decrease in strength because the pores act as places for crack initiation which lead to a decrease in strength. In this way, the Mix 7 has the highest strength and Mix 4 has the lowest strength, and its strength is half of the strength of Mix 7 (30 MPa). The strength of Mix 5, 8, and 6 was measured to be 23, 22 and 18 MPa, respectively.

As the firing temperature increases to 1100 °C, according to Fig. 14, the porosity of all samples increases resulting in a decrease in strength. This is true for all samples except Mix 4. In other words, without considering Mix 4, the order of the strength of the other mixtures is according to their percentage of apparent porosity, and thus, Mix 7, 5, 8 and 6 have the highest to lowest strength respectively with the amounts of 19, 15, 14 and 11 MPa. The remarkable thing about Mix 4 is that despite the fact that this mixture has the highest amount of apparent porosity according to Fig. 14, it also shows the highest amount of strength for the samples fired at 1100 °C (20 MPa). In fact, all the points that were mentioned earlier regarding the amount of porosity are also true for this mixture, but another parameter has played a role in this temperature and in this mixture, which has an effect greater than the effect of the amount of porosity, and has overcome it and led to increased strength. This parameter is the formation of sillimanite phase in Mix 4 at this temperature. This phase has been identified in this mixture according to Fig. 3. The sillimanite phase is the phase that leads to an increase in strength^42,43^. In fact, this phase is not detected in the rest of the samples, and for this reason, this increase in strength at this temperature is not observed for the rest of the mixtures. Also, the reason for the formation of sillimanite in Mix 4 can be related to its amount of silica, which can be formed with the appropriate amount of silica and compliance with the stoichiometric ratio of this phase. Furthermore, the presence of gehlenite in all samples (according to Fig. 3), results in the reduction in the strength of all samples generally^44^. However, the mentioned factors (apparent porosity and sillimanite phase) are more effective and they determine the strength trends of the samples.

By increasing the firing temperature to 1550 °C, the strength of all samples increases remarkably. As seen in Fig. 14, at 1550 °C, the porosity of the samples decreases due to the reasons mentioned earlier. At this temperature, the apparent porosity of the mixtures is almost equal to their porosity at 110 °C. Therefore, at 1550 °C, if only the porosity factor plays a role in determining the strength of the samples, the strength of the mixtures should be about the same as their strength at 110 °C; but as can be seen, the strength of the samples at 1550 °C is much higher than 110 °C. Considering this issue, it can be understood that formation of new phases at this firing temperature plays an important role in increasing the strength of these samples.

By examining the X-ray diffraction patterns, it can be seen that at 1550 °C, except for corundum, CA_6_ phase was formed in all samples. The structure of the sample matrix is reinforced with CA_6_ phase particles and leads to an increase in strength^45^. Therefore, this phase is one of the important factors in increasing the strength at 1550 °C. On the other hand, considering that the type and amount of cement used in all samples is the same and equal, therefore, the amount of calcia in all samples is also almost equal. Also, there is sufficient amount of alumina. Therefore, it can be said that the amount of CA_6_ phase, which is a combination of alumina and calcia, is almost the same in all samples. From all the mentioned cases, it can be concluded that although the CA_6_ phase is a very effective phase in increasing the strength of the samples at this temperature, there is another factor or, in better words, formation of another phase at this temperature that leads to the difference in the order of the strength of the samples fired at this temperature compared to 1100 °C. In other words, if only considering the increase in strength based on the CA_6_ phase, due to the almost identical amount of this phase in all mixtures, the order of the strength of the mixtures should be similar to the trend seen at 1100 °C, while it is different.

According to the electron microscope images and EDX point analysis, in addition to corundum and CA_6_ phases, another phase consisting of alumina, silica, and calcia can be found in the sample fired at 1550 °C. This phase was not detected by X-ray diffraction which indicated non-crystalline nature of this phase. The presence of this amorphous phase has led to an increase in the strength of the samples fired at 1550 °C. However, the amount of increase in strength for different samples depends on the amount of formation of this phase in such sample. A sample that contains more silica has a higher amount of this phase and as a result experiences a higher strength. Therefore, Mix 4, which has the highest amount of silica (based on Table 4), has the highest strength (80 MPa), and on the other hand, Mix 7, with the lowest amount of silica (based on Table 4), has the lowest strength (56 MPa). The rest of the mixtures are also dependent on the amount of silica, they have strengths between Mix 4 and Mix 7, so that Mix 6, 8 and 5, which respectively have the highest to the lowest amount of silica (based on Table 4), have strengths of 74, 67 and 62 MPa, respectively.

It should be noted that the strengths obtained for the samples fired at 1550 °C are room temperature values. Therefore, detrimental effect of low melting point phases within the structure should be taken into account when the refractory is going to be used at temperatures higher than 1400 °C.

## Conclusions

From the results of this study it can be concluded that:

1. The mixtures containing carboxymethyl cellulose and 1% of H19 were unfavorable in terms of rheological properties, but other mixtures were suitable in this regard.
2. According to X-ray diffraction analysis and EDX point analysis, it was found that for the samples heated at 110 °C, quartz and C_3_AH_6_ phases had formed. For all the samples fired at 1100 °C, gehlenite was formed whereas, sillimanite is only found in the sample containing 2% of H19. For the samples fired at 1550 °C, CA_6_ and the amorphous phases were formed. It should also be noted that at all these temperatures, the main phase formed is the corundum phase.
3. At all temperatures, Mix 7 can be chosen due to its highest density and lowest apparent porosity; especially at 110 °C, which also has the highest strength.
4. At 1100 °C, Mix 4 can be used because it has the highest strength.
5. At 1550 °C, Mix 4 has the highest strength and should be selected and used, but due to the presence of low melting point phases in this mixture, the use of this mixture is not recommended and depending on the type of application, other mixtures with less silica can be used.

## Data Availability

The datasets used and/or analysed during the current study available from the corresponding author on reasonable request.
